# Case Report: delayed recognition of pulmonary tuberculosis during immunosuppression for suspected connective tissue disease

**DOI:** 10.3389/fmed.2026.1905687

**Published:** 2026-07-28

**Authors:** Zheng Yang, Yuhe Yan, Mengyi Zheng

**Affiliations:** 1 Department of Infectious Diseases, Quzhou Second People’s Hospital, Quzhou, Zhejiang, China; 2 Graduate School of Shanxi Medical University, Taiyuan, Shanxi, China; 3 Department of Rheumatology and Immunology, Quzhou Second People’s Hospital, Quzhou, Zhejiang, China

**Keywords:** connective tissue disease, delayed diagnosis, diagnosis, immunosuppression, tuberculosis

## Abstract

**Background:**

Pulmonary tuberculosis may be difficult to recognize in patients receiving immunosuppression for suspected connective tissue disease, because clinical symptoms, inflammatory markers, pulmonary abnormalities, and imaging findings may be attributed to autoimmune disease activity.

**Case presentation:**

A 57-year-old woman with dry mouth, myalgia, arthralgia, and positivity for antinuclear antibody (ANA) and anti-SSA/SSB antibodies was initially considered to have Sjögren syndrome or undifferentiated connective tissue disease. She sequentially received glucocorticoids, belimumab, tocilizumab, and baricitinib. During treatment, she developed recurrent low-grade fever, marked weight loss, pulmonary nodules and consolidation, mediastinal lymphadenopathy, and painful subcutaneous nodules. Three TB-related T-cell immunological assays were negative, including two interferon-gamma release assay tests and one T-SPOT. TB assay. Biopsy of a subcutaneous lesion showed acute and chronic inflammation with suspected granuloma formation, and next-generation sequencing of the tissue did not identify a causative pathogen. Because fever persisted and respiratory symptoms subsequently developed, an infectious etiology was reconsidered, and respiratory microbiological testing was repeated. The sputum acid-fast bacilli smear was positive (3+), *Mycobacterium tuberculosis* complex nucleic acid testing showed a high DNA load, molecular resistance testing demonstrated rifampicin susceptibility, and sputum mycobacterial culture was subsequently positive. Anti-TB treatment with a 2HRZE/10HRE regimen was followed by defervescence, regression of the panniculitis-like lesions, smear conversion, culture conversion, and radiological improvement.

**Conclusion:**

Negative IGRA-related tests should not be used to exclude active TB in immunosuppressed patients. Persistent fever, weight loss, pulmonary lesions, lymphadenopathy, or granulomatous inflammation should prompt repeat microbiological testing before escalation of immunosuppression.

## Introduction

Tuberculosis (TB) remains a leading cause of infectious morbidity and mortality worldwide. A recent Global Burden of Disease analysis estimated 9.40 million incident TB cases and 1.35 million TB-related deaths in 2021, underscoring the persistent burden of TB despite progress in control strategies (1). The diagnosis of pulmonary TB depends on clinical features, radiological abnormalities, and microbiological evidence, including acid-fast bacilli (AFB) smear, mycobacterial culture, and nucleic acid amplification testing. However, pulmonary TB has varied clinical presentations, and cases without typical symptoms or classic imaging findings are more likely to be recognized late (2, 3).

Patients with rheumatic diseases face an increased risk of active TB due to the coexistence of disease-related immune dysregulation and treatment-related immune suppression. In Chinese cohorts with rheumatic disease, active TB has been associated with the specific disease category, glucocorticoid exposure, a previous history of TB, and overall disease activity (4, 5). Disease-specific studies in systemic lupus erythematosus and Takayasu arteritis further support the need for longitudinal TB risk assessment in patients with autoimmune inflammatory diseases (6, 7).

Biologic disease-modifying antirheumatic drugs and targeted synthetic agents can further influence TB risk. Rheumatoid arthritis cohort studies have evaluated TB occurrence during treatment with biologic agents, tocilizumab, and Janus kinase inhibitors, and the observed risk varies by drug class and local TB burden (8–12). Belimumab has shown an acceptable safety profile in systemic lupus erythematosus studies, but the infection risk remains relevant when the drug is combined with glucocorticoids or other immunosuppressive agents (13, 14).

Interferon-gamma release assays (IGRAs), including the T-SPOT. TB test, are commonly used as adjunctive tests for TB infection. These assays measure host T-cell responses to MTB-specific antigens rather than directly detecting the organism. False-negative IGRA or T-SPOT. TB results can occur in active TB, particularly in older patients and those with lymphopenia, connective tissue disease, or impaired T-cell function (15, 16). The extent of pulmonary disease on chest CT has also been associated with false-negative T-SPOT. TB results, highlighting the need to interpret negative immune-based tests in clinical context (17, 18). We report a patient in whom pulmonary TB was diagnosed only after repeated microbiological evaluation, despite multiple negative TB-related T-cell immunological tests and panniculitis-like soft-tissue manifestations that developed during immunosuppression for suspected connective tissue disease.

## Case presentation

In 2024, a 57-year-old woman developed diffuse myalgia, fatigue, dry mouth, dry eyes, dysphagia, and polyarthralgia. Initial evaluation at outside hospitals showed positive antinuclear antibodies, anti-SSA/Ro52, anti-SSA/Ro60, and anti-SSB antibodies, mildly elevated creatine kinase, alongside a and multiple small pulmonary nodules on chest CT. Sjögren syndrome or undifferentiated connective tissue disease was suspected, and hydroxychloroquine therapy was initiated with limited clinical improvement.

In July 2025, she experienced an unintentional weight loss of approximately 20 kg, alongside recurrent shoulder pain, headaches, a sore throat, and difficulty raising both upper limbs. A left supraclavicular lymph node biopsy demonstrated reactive hyperplasia. Autoimmune disease activity was suspected, and her treatment included hydroxychloroquine, belimumab, and systemic glucocorticoids. Although her pain improved, transiently recurrent low-grade fever persisted, reaching a maximum temperature of approximately 38.8 °C and accompanied by elevated inflammatory markers.

TB-related T-cell immunological testing was repeatedly negative. Whole-blood IFN-gamma release assay-type tests performed on July 30 and September 29, 2025, were negative with preserved positive-control responses, during her hospitalization at a tertiary rheumatology center; a T-SPOT. TB test performed on November 22, 2025, was again negative, with an antigen-stimulated spot count of 3, a nil control of 0, and a positive control of at least 20. These negative results did not support TB at that stage, although they could not exclude active disease (Table 1).

**Table 1:** Chronological summary of the diagnostic course and treatment response.

Time	Key clinical/Laboratory findings	Clinical interpretation or intervention
2024	Sicca symptoms, myalgia, arthralgia, ANA/anti-SSA/anti-SSB positivity, and small pulmonary nodules on chest CT.	Sjögren’s syndrome or UCTD was suspected; hydroxychloroquine was initiated.
Jul-25	Weight loss of approximately 20 kg, shoulder pain, headache, sore throat, reactive supraclavicular lymph node, and negative IGRA-type test.	Autoimmune disease activity was suspected; hydroxychloroquine, belimumab, and glucocorticoids were used.
Sep-25	Persistent fever and inflammatory activity; repeat IGRA-type test was negative.	TB was not supported by immunological testing; infection remained unconfirmed.
Oct-25	PET/CT showed FDG-avid muscles and soft tissues, small pulmonary nodules, and mediastinal/hilar lymphadenopathy.	Inflammatory or autoimmune activity was suspected; tocilizumab was given, with transient improvement.
Nov–Dec 2025	Painful panniculitis-like subcutaneous indurations; HRCT showed pulmonary consolidation, nodules, and mediastinal lymphadenopathy; MRI/ultrasound showed soft-tissue edema; T-SPOT. TB was negative; biopsy showed acute/chronic inflammation with suspected granuloma; tissue mNGS was negative.	UCTD-related inflammation and panniculitis-like lesions were considered; glucocorticoids and baricitinib were used.
17-Jan-26	Sputum AFB smear was positive (3+); MTB complex DNA load was high; rifampicin susceptibility was reported.	Active pulmonary TB was diagnosed; 2HRZE/10HRE was started.
29-Jan-26	Sputum mycobacterial culture was positive.	Microbiological confirmation was obtained.
21-Feb-26	Sputum AFB smear converted to negative.	Early microbiological response.
21-Mar-26	Follow-up chest CT showed absorption of pulmonary lesions.	Radiological response.
30-Apr-26	Sputum mycobacterial culture converted to negative.	Culture conversion.

In October 2025, a PET/CT scan demonstrated diffuse fluorodeoxyglucose uptake in multiple muscle and soft-tissue regions, together with small pulmonary nodules and mediastinal/hilar lymphadenopathy. These findings were interpreted as indicative of inflammatory or autoimmune disease activity. Tocilizumab was administered, and her fever and pain temporarily improved.

**Figure 1:**
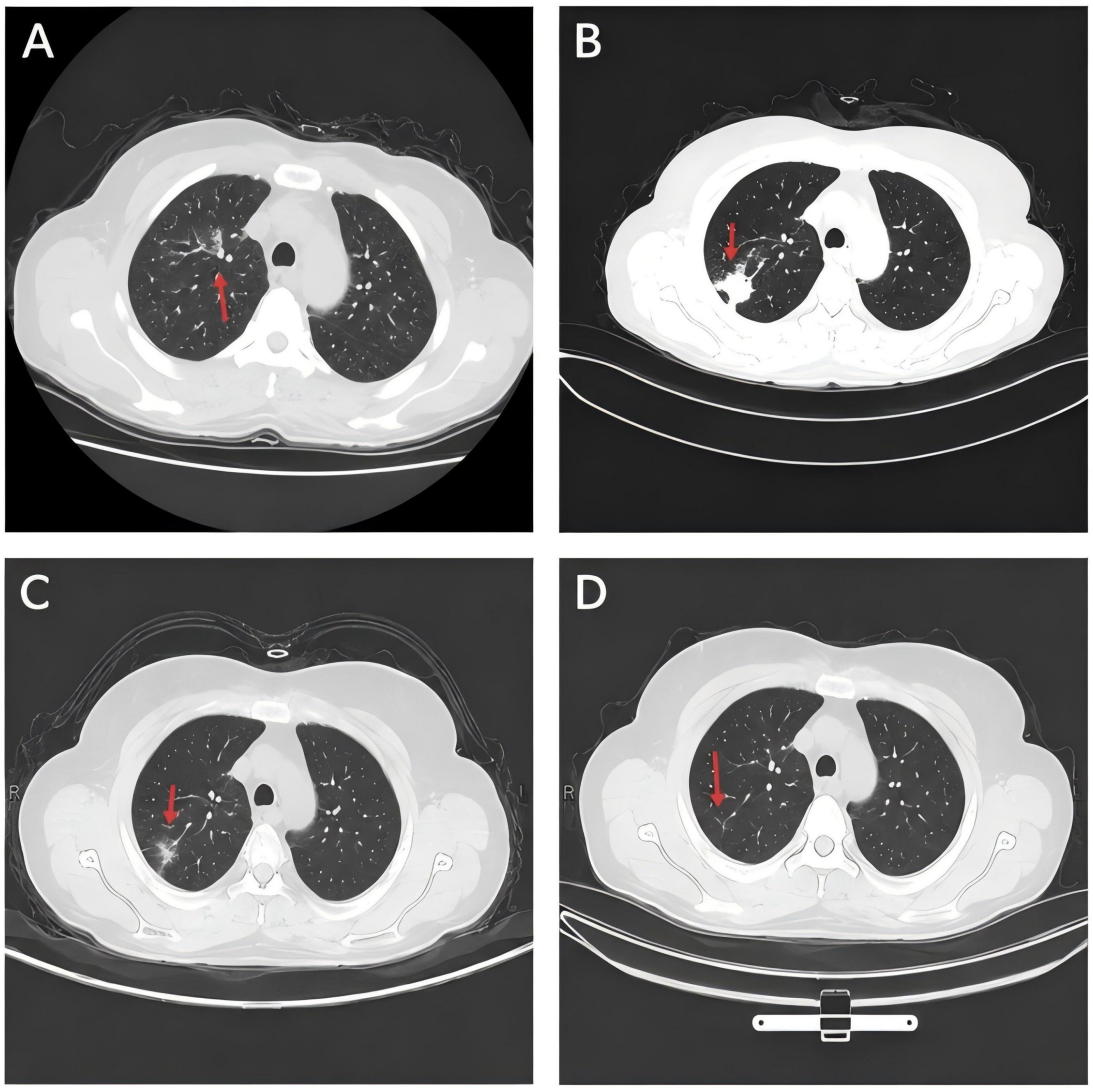
Serial chest CT findings. **(A)** Chest CT on July 10, 2025, showed scattered small pulmonary nodules without overt right upper-lobe consolidation. **(B)** HRCT on November 20, 2025, demonstrated right upper-lobe consolidation/exudative opacity with scattered bilateral small nodules. **(C)** Chest CT on January 15, 2026, shortly before microbiological confirmation, showed persistent right upper-lobe exudative lesions. **(D)** Follow-up chest CT on March 21, 2026, after anti-tuberculosis treatment showed interval absorption of the right upper-lobe lesion.

In November 2025, she developed generalized pruritus and new painful panniculitis-like subcutaneous indurations over the right back and left upper arm, with local warmth and restricted shoulder movement. She was hospitalized in the rheumatology department from November 20 to December 13, 2025. Serial chest CT scans demonstrated radiological progression during the diagnostic course. Compared with the earlier CT in July 2025, HRCT on November 20, 2025, showed right upper-lobe consolidation/exudative opacity, a right lower-lobe solid nodule, scattered bilateral micronodules, and multiple mediastinal lymph nodes. A follow-up CT scan was obtained before microbiological confirmation showed persistent right upper-lobe exudative lesions, whereas a CT scan was obtained after anti-tuberculosis therapy demonstrated interval absorption of the pulmonary lesions (Figure 1). Chest magnetic resonance imaging and soft-tissue ultrasonography further revealed multifocal subcutaneous and muscular edema of the chest wall and back, with partial necrosis and liquefaction, supporting the presence of concomitant panniculitis-like soft-tissue involvement.

Biopsy of the right posterior back lesion revealed mild perivascular inflammatory cell infiltration in the dermis and focal fibrous proliferation in the subcutaneous adipose tissue, with acute and chronic inflammation and suspected granuloma formation. No malignancy was identified. Tissue metagenomic next-generation sequencing was negative. The working diagnosis remained connective tissue disease-related panniculitis-like inflammation with pulmonary abnormalities. She received glucocorticoids, empirical antibiotics, supportive therapy, and then baricitinib. Fever and subcutaneous indurations improved transiently before discharge.

**Figure 2:**
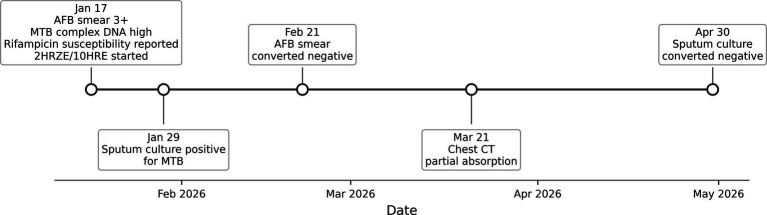
Microbiological confirmation and treatment response timeline. AFB, acid-fast bacilli; MTB, *Mycobacterium tuberculosis*; HRZE, isoniazid, rifampicin, pyrazinamide, and ethambutol; HRE, isoniazid, rifampicin, and ethambutol.

After discharge, fever recurred and cough with sputum gradually developed. Given the persistent constitutional symptoms, marked weight loss, pulmonary lesions, mediastinal/hilar lymphadenopathy, suspected granulomatous inflammation, and relapse after immunosuppression, an infectious etiology was reconsidered. On January 17, 2026, sputum AFB smear was positive (3+). MTB complex nucleic acid testing showed a high DNA load, and molecular resistance testing reported rifampicin susceptibility. Sputum mycobacterial culture was reported positive on January 29, 2026. Active pulmonary TB was diagnosed. The microbiological confirmation and early treatment course are summarized in Figure 2.

**Figure 3:**
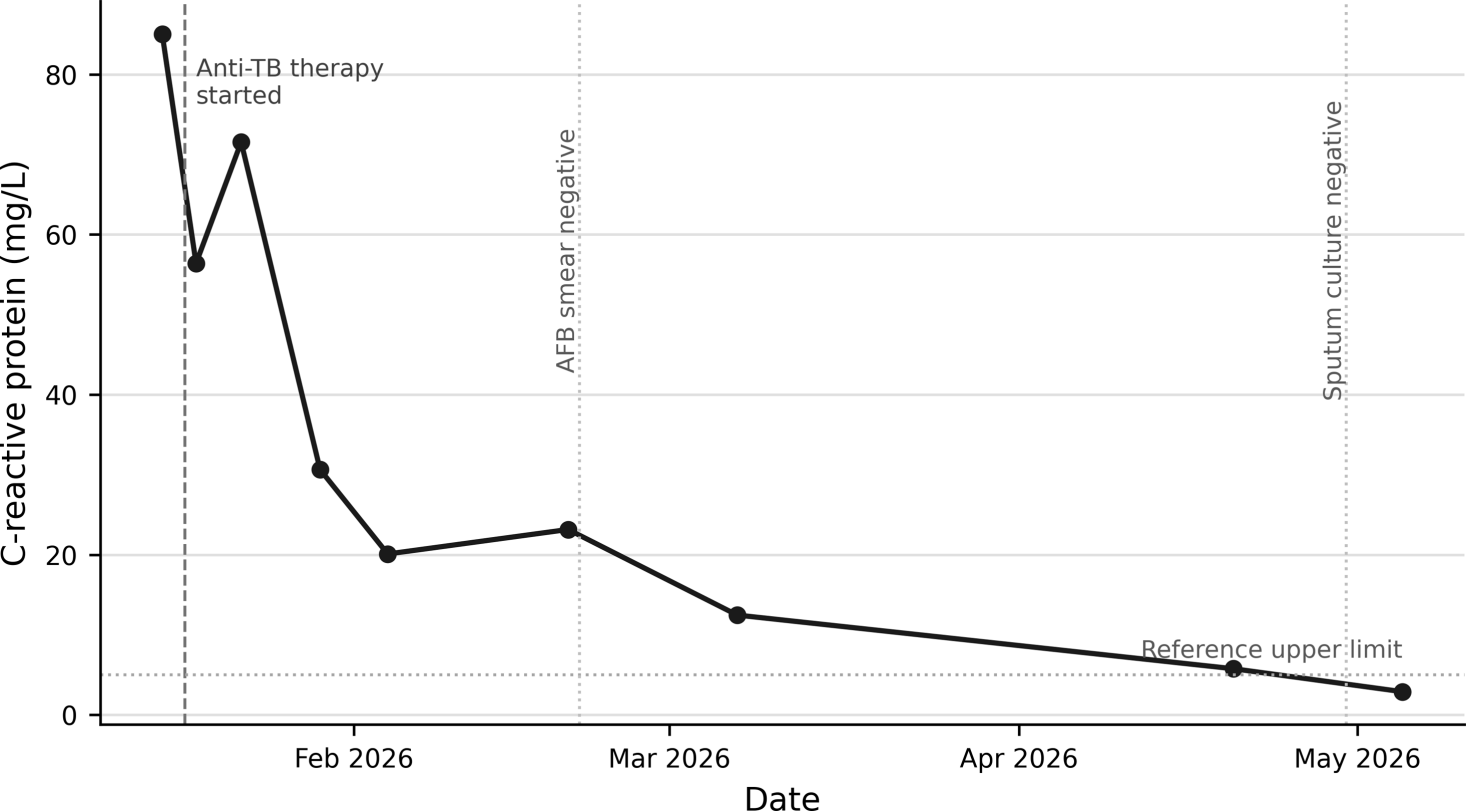
C-reactive protein trend after initiation of anti-tuberculosis therapy. The vertical markers indicate anti-tuberculosis treatment initiation, smear conversion, and sputum culture conversion.

After TB was diagnosed, immunosuppressive therapy was de-escalated by the treatment team. On January 17, 2026, anti-TB treatment was initiated with a 2HRZE/10HRE regimen: a 2-month intensive phase with isoniazid, rifampicin, pyrazinamide, and ethambutol, followed by a 10-month continuation phase with isoniazid, rifampicin, and ethambutol. The patient also had steroid-induced diabetes. Within 1 week of anti-TB therapy, the panniculitis-like indurations and edema of the face, back, muscles, and subcutaneous tissue improved. Fever and respiratory symptoms subsequently resolved. Sputum AFB smear converted negative on February 21, 2026; chest CT on March 21, 2026, showed absorption of pulmonary lesions; and sputum mycobacterial culture converted negative on April 30, 2026. The inflammatory response after anti-TB therapy is shown in Figure 3.

## Discussion

This case highlights a diagnostic challenge in pulmonary TB recognition during immunosuppression for suspected connective tissue disease. The initial diagnosis was clinically plausible because the patient presented with sicca symptoms, myalgia, arthralgia, and autoantibody positivity. The later combination of persistent fever marked weight loss, pulmonary lesions, lymphadenopathy, panniculitis-like subcutaneous indurations, and suspected granulomatous inflammation should prompt a renewed consideration of chronic infection, particularly TB.

Cumulative immunosuppression likely contributed to these diagnostic challenges. The control of MTB depends on coordinated T-cell and macrophage responses and the maintenance of granulomatous inflammation (19). Glucocorticoid exposure has been associated with active TB in rheumatic disease cohorts, and autoimmune disease activity may further increase susceptibility (4, 6). Biologic and targeted synthetic agents can alter cytokine pathways relevant to host defense, although the associated TB risk differs by drug class and clinical setting (9–12). This case should not be interpreted as implicating any single drug as the cause of the patient’s TB. Rather, her sequential exposure to glucocorticoids, belimumab, tocilizumab, and baricitinib created a clinical setting in which TB progression, attenuated inflammatory signs, and reduced reliability of immune–response-based testing could coexist.

The clinical course also illustrates attribution bias. Fever, fatigue, myalgia, arthralgia, elevated inflammatory markers, and FDG-avid soft-tissue lesions were repeatedly interpreted within an autoimmune framework. The transient clinical response after tocilizumab administration was potentially misleading. Tocilizumab can suppress C-reactive protein responses during serious infection, and retrospective studies have noted that infection may be under-recognized when acute-phase reactants are blunted (20, 21). Transient improvement after anti-inflammatory therapy should therefore not be used as evidence against TB when systemic symptoms and pulmonary abnormalities persist. Repeatedly negative TB-related T-cell testing added a second source of diagnostic uncertainty. IGRAs and the T-SPOT. TB measures interferon-gamma release by host T cells after stimulation with MTB-specific antigens; it does not directly detect the organism. False-negative results have been reported in active TB, with age, host comorbidity, connective tissue disease, anemia, lymphopenia, and disease distribution identified as relevant factors (15, 22). In this patient, repeated immunosuppression and lymphopenia provide a biologically plausible explanation for the negative tests. Radiological findings represented another diagnostic challenge. The patient did not present with classic cavitary pulmonary TB or clear bronchogenic spread. Instead, CT imaging showed small nodules, patchy opacities, consolidation or exudation, and mediastinal or hilar lymphadenopathy. Pulmonary TB can be grouped into heterogeneous clinical–radiological phenotypes, and atypical cases lacking typical clinical features may experience diagnostic delay (2, 3). In such settings, the longitudinal clinical pattern is more informative than a single CT morphology.

The panniculitis-like lesions require cautious interpretation. Their rapid improvement after anti-TB therapy supports an association with active TB, and the suspected granulomatous inflammation on biopsy increased concern for chronic infection. However, tissue mNGS was negative, and no direct mycobacterial evidence was obtained from the subcutaneous lesion. Cutaneous tuberculosis and nontuberculous mycobacterial infections can show diverse clinicopathological patterns, and tuberculous panniculitis-like presentations have been reported (23, 24). Nevertheless, the evidence in this case supports a diagnosis of pulmonary TB associated with panniculitis-like subcutaneous indurations, but not definite cutaneous or soft-tissue TB.

In clinical practice, negative IGRA results should not end the microbiological evaluation when clinical suspicion persists in an immunosuppressed patient. Repeated sputum AFB smear, mycobacterial culture, and MTB nucleic acid testing should be pursued. Bronchoscopy, lymph node sampling, or tissue testing should be considered when sputum is unavailable or negative despite a high clinical suspicion. Recent diagnostic accuracy studies support the clinical value of the Xpert MTB/RIF Ultra assay for the rapid detection of MTB and rifampicin resistance, particularly when early microbiological confirmation is needed (25, 26). In this case, the diagnosis was established only after direct respiratory microbiological testing, and the patient’s treatment response was supported by symptom resolution, smear conversion, sputum culture conversion, and radiological improvement.

This report has several limitations. First, although rifampicin susceptibility was reported by molecular testing, full phenotypic drug susceptibility testing was unavailable. Second, the panniculitis-like lesions lacked direct mycobacterial confirmation. Third, a single case cannot determine the independent contribution of each immunosuppressive agent to TB onset or its atypical presentation. Despite these limitations, the microbiological evidence and the temporal response to anti-TB therapy support active pulmonary TB as the final diagnosis explaining the later clinical course.

## Data Availability

The original contributions presented in the study are included in the article/supplementary material, further inquiries can be directed to the corresponding author.
